# Supplementary material for: Companion Animal Ownership and Mood States of University Students Majoring in Animal Sciences during the COVID-19 Pandemic in Japan
Source: Animals (Basel). 2021 Oct 3;11(10):2887. doi: 10.3390/ani11102887 (PMC8532765; doi:10.3390/ani11102887)
Supplement: Supplementary file 1 [file animals-11-02887-s001.zip › animals-1300274-supplementary.pdf]

The impact of COVID-19 on young people

This questionnaire is part of my graduation research project. Its purpose is to understand how you spend your days and what your mood is under the stay-at-home request from the government due to the COVID-19 pandemic. This questionnaire should take no more than 10–15 minutes to complete. Your participation is voluntary, and whether or not you answer this questionnaire will not affect your grade. The document is anonymous, and your participation in this survey is considered as your consent to this study. If you have any questions, please feel free to contact us at:

Department of Animal Sciences, Teikyo University of Science.

- Daiki Namekata（s17ag108@st.ntu.ac.jp）
- Supervisor: Mariko Yamamoto（yamamoto-mariko@ntu.ac.jp）

Q1. Gender

1. Male 2. Female 3. No answer

Q2. Age

（　　）years old

Q3. Have you temporarily returned home due to the effects of COVID-19?

1. Yes, I temporarily returned home.
2. No, I had already returned home for the spring break.
3. No, I have always lived with my family.
4. No, I have not returned home.
5. Other (　　　　　　　　　　　　　　　　)

Q4. Do you currently live alone, or do you live with other people? If you have returned home temporarily due to the effects of COVID-19, please select which answer best describes your return home.

1. I live alone.
2. I live with my family member(s).
3. I live with my sibling(s).

4. I live with my friend(s).
5. Other (   )

Q5-1. Are you currently experiencing difficulties due to the effects of COVID-19?

1. I am not experiencing difficulty.
2. I am experiencing slight difficulty.
3. I am experiencing moderate difficulty.
4. I am experiencing great difficulty.

Q5-2. Choose all of the options below that apply to how you relieve stress.

1. Watching TV
2. Watching movies
3. Reading books and comics
4. Watching online videos
5. Checking SNS
6. Playing games
7. Playing with a pet
8. Sleeping
9. Talking with family members (including phone calls)
10. Phone calls with friends
11. Exercising
12. Other (   )

Below is a list of words that describe some feelings people have. Read each one carefully and choose the number that best describes how you felt in the past week, including today.

0. Not at all
1. A little
2. Moderately
3. Quite a bit
4. Extremely

1. Friendly 0 1 2 3 4

2. Tense	0	1	2	3	4
3. Angry	0	1	2	3	4
4. Worn out	0	1	2	3	4
5. Lively	0	1	2	3	4
6. Confused	0	1	2	3	4
7. Considerate of others	0	1	2	3	4
8. Sad	0	1	2	3	4
9. Positive	0	1	2	3	4
10. Grouchy	0	1	2	3	4
11. Energetic	0	1	2	3	4
12. Cannot remain calm	0	1	2	3	4
13. Hopeless	0	1	2	3	4
14. Anxious	0	1	2	3	4
15. Unable to concentrate	0	1	2	3	4
16. Tired	0	1	2	3	4
17. Feel useful to others	0	1	2	3	4
18. Nervous	0	1	2	3	4
19. Miserable	0	1	2	3	4
20. Have trouble thinking clearly	0	1	2	3	4
21. Furious	0	1	2	3	4
22. Bushed	0	1	2	3	4
23. Worrisome	0	1	2	3	4
24. Warm toward others	0	1	2	3	4
25. Helpless	0	1	2	3	4
26. Weary	0	1	2	3	4
27. Bewildered	0	1	2	3	4
28. Resentful	0	1	2	3	4
29. Trusting of others	0	1	2	3	4
30. Bad-tempered	0	1	2	3	4
31. Worthless	0	1	2	3	4
32. Vigorous	0	1	2	3	4

33. Uncertain about things 0 1 2 3 4

34. Exhausted 0 1 2 3 4

35. Full of pep 0 1 2 3 4

Q6. Do you currently have a pet? If you have returned home temporarily, please indicate whether or not there is a pet in your current home.

1. Yes
2. No

For those who chose “2. No,” this was the last question. Thank you for your cooperation.

【Below are questions for those who chose “1. Yes.”】

Q7-1. What kind of animal do you have? (Please list all animals you have.)

Example: Dog, cat, etc.

Q7-2. Below asks you about your usual relationship with the animal you were thinking of in Q7-1. To what extent do the following descriptions apply to you? Please choose the applicable number.

1. Not true.
2. Somewhat not true
3. Neither
4. Somewhat true
5. True

Please answer without thinking too much. “CA” means companion animal, or pet. Please answer about the animal you choose as the most important to you.

1. My CA understands me better than anyone else. 1 2 3 4 5
2. When I am in trouble or suffering, it is comforting to thinking about my CA.

1 2 3 4 5

3 . I can tell my CA what I cannot tell others. 1 2 3 4 5

4 . It makes me feel calm just knowing my CA is there. 1 2 3 4 5

5 . I feel at ease being with my CA. 1 2 3 4 5

6 . I go near my CA when I have a problem or feel sad. 1 2 3 4 5

7 . I talk to my CA when something bad happens. 1 2 3 4 5

8 . I go near my CA when I have fun or happy things. 1 2 3 4 5

9 . I like spending time with my CA. 1 2 3 4 5

10. My CA makes me feel happy just by looking at. 1 2 3 4 5

11. I go out more often because I have a CA. 1 2 3 4 5

12. My CA makes it easier to talk with people of other generations (different ages). 1 2 3 4 5

13. I have become more involved with my neighbors since I have my CA. 1 2 3 4 5

14. Strangers talk to me while I am walking my CA. 1 2 3 4 5

15 Through my CA, I have become acquainted with people of various generations, ages, and positions. 1 2 3 4 5

16. Talking about my CA is one way to communicate with someone you don't get along with. 1 2 3 4 5

17. I feel close to other people who have a CA. 1 2 3 4 5

18. The presence of my CA has reduced family quarrels. 1 2 3 4 5

19. The story of my CA is a central topic of conversation in my family. 1 2 3 4 5

20. My CA has increased the topic of conversation in my family. 1 2 3 4 5

21. My family is united because of our CA. 1 2 3 4 5

22. I seek out my CA before going to anyone in my family when I'm feeling stressed. 1 2 3 4 5

23. I learned to care for something weaker than myself by having my CA. 1 2 3 4 5

24. I have learned how to take care of something by having my CA. 1 2 3 4 5

25. I have a feeling of satisfaction that I am raising a living being. 1 2 3 4 5

26. I am happy when my CA receives a compliment from someone. 1 2 3 4 5

27. I often pet my CA. 1 2 3 4 5

28.	My CA makes me feel like I am needed.	1	2	3	4	5
29.	My CA makes me feel safe.	1	2	3	4	5
30.	My CA gives enjoyment and makes me laugh.	1	2	3	4	5
31.	I feel calm by petting my CA.	1	2	3	4	5
32.	I enjoy watching my CA.	1	2	3	4	5
33.	My CA makes me feel loved.	1	2	3	4	5
34.	My CA makes me feel trusted.	1	2	3	4	5

Thank you for your cooperation.

Department of Animal Sciences, Teikyo University of Science.

- Daiki Namekata（s17ag108@st.ntu.ac.jp）
- Supervisor: Mariko Yamamoto（yamamoto-mariko@ntu.ac.jp）
